# NF‐κB‐mediated inflammatory damage is differentially affected in SH‐SY5Y and C6 cells treated with 27‐hydroxycholesterol

**DOI:** 10.1002/fsn3.1005

**Published:** 2019-04-11

**Authors:** Wei‐Wei Ma, Chao‐Qun Li, Lei Zhao, Yu‐Shan Wang, Rong Xiao

**Affiliations:** ^1^ Beijing Key Laboratory of Environmental Toxicology, School of Public Health Capital Medical University Beijing China; ^2^ Department of Molecular Physiology and Biophysics, Holden Comprehensive Cancer Center University of Iowa Carver College of Medicine Iowa City Iowa

**Keywords:** 27‐hydroxycholesterol, C6 cells, inflammatory damage, nuclear factor‐κB, SH‐SY5Y cells

## Abstract

Previous studies have demonstrated that 27‐hydroxycholesterol (27‐OHC), a cholesterol metabolite, was involved in the inflammatory process of Alzheimer's disease (AD). The present study aimed to investigate the 27‐OHC‐induced inflammatory damage to neurons and astrocytes and the underlying mechanism(s) accounting for this damage. Human neuroblastoma cells (SH‐SY5Y cells) and rat glioma cells (C6 cells) were treated with vehicle or 27‐OHC (5, 10, or 20 μM) for 24 hr. The levels of secreted interleukin‐1β (IL‐1β), interleukin‐10 (IL‐10), tumor necrosis factor alpha (TNF‐α), and inducible nitric oxide synthase (iNOS) were determined by using an enzyme‐linked immunosorbent assay (ELISA). Immunofluorescence staining was used to determine the cellular expression of toll‐like receptor 4 (TLR4) and transforming growth factor‐β (TGF‐β). The mRNA and protein expression levels of nuclear factor‐κB p65 (NF‐κB p65), nuclear factor‐κB p50 (NF‐κB p50) and cyclooxygenase‐2 (COX‐2) in both SH‐SY5Y and C6 cells were also detected by real‐time PCR and Western blot, respectively. The results of this study showed that 27‐OHC treatment increased secretion of TNF‐α and iNOS and decreased secretion of IL‐10, upregulated expression of TGF‐β, NF‐κB p65 and p50, and downregulated expression of COX‐2 in SH‐SY5Y cells. In C6 cells, treatment with 27‐OHC resulted in decreased secretion of IL‐1β, IL‐10, TNF‐α, and iNOS, and increased expression of TLR4 and TGF‐β. These results suggest that 27‐OHC may cause inflammatory damage to neurons by activating the TGF‐β/NF‐κB signaling pathway and to astrocytes by activating the TLR4/TGF‐β signaling, which results in the subsequent release of inflammatory cytokines.

## 1 INTRODUCTION

Alzheimer's disease (AD) is a debilitating neurodegenerative disease, with 35 million reported cases worldwide (Konietzko, 2015). Over the past few years, mounting evidence has implicated sustained glial‐mediated inflammation as a major contributor to the neurodegenerative processes and cognitive deficits observed in AD (Fakhoury, 2018; Forloni & Balducci, 2018; Laurent, Buee, & Blum, 2018). Excessive activation of astrocytes and microglia by different inflammatory factors is closely associated with the occurrence and development of AD (Serrano‐Pozo et al., 2013). Glial cells can be activated by accumulation of β‐amyloid peptide (Aβ), which contributes to nerve fiber entanglement and leads to the typical pathological changes in AD. In animal‐based experiments, upregulation of anti‐inflammatory factors has been shown to suppress excessive expression of the amyloid precursor protein (APP) in transgenic mice (Ma et al., 2018). Additionally, our previous studies showed that microglia and astrocytes that were damaged by β‐amyloid peptide 25–35 (Aβ25–35) could release inflammatory factors such as interleukin‐1 (IL‐1), interleukin‐6 (IL‐6), and tumor necrosis factor (TNF‐α) (Ma, Ding et al., 2015; Zhou et al., 2014). These results suggest that the release of inflammatory cytokines in neurons and astrocytes is closely associated with the level of Aβ and eventually leads to the development of AD (Fang, Sun, Zhou, Qiu, & Peng, 2018).

Increasing numbers of studies have suggested that 27‐hydroxycholesterol (27‐OHC), an oxidative metabolite of cholesterol, may cause inflammation in the brain and is associated with an increased risk of dementia including AD (Dias, Brown, Shabir, Polidori, & Griffiths, 2018; Testa et al., 2014,2016). Our recent studies showed the toxic effect of 27‐OHC in C6 glioma cells and in the brains of Sprague–Dawley rats fed a high‐cholesterol diet. Our results suggest that 27‐OHC may be able to inhibit cholesterol synthesis in the brain (An et al., 2017; Zhang et al., 2018). In addition to being involved in cholesterol metabolism, 27‐OHC is also involved in the inflammatory process. Gargiulo et al. (2015) demonstrated that 27‐OHC could enhance the release of IL‐1β and TNF‐α through a nuclear factor‐κB (NF‐κB)/toll‐like receptor 4 (TLR4)‐dependent pathway in human promonocytic U937 cells. The expression of NF‐κB and TNF‐α was upregulated in retinal pigment epithelial cells treated with 27‐OHC at concentrations between 10 and 20 μM (Prasanthi et al., 2009). An additional study reported that 27‐OHC significantly increased the levels of NF‐κB and transcription factor binding to β‐site amyloid precursor protein cleaving enzyme 1 (BACE1), which in turn increased the formation of Aβ and the transcription of BACE1 in SH‐SY5Y cells (Marwarha, Raza, Prasanthi, & Ghribi, 2013).

Accumulating evidence suggests that 27‐OHC may be involved in the inflammatory processes that contribute to the development of AD (Testa et al., 2014,2016). However, few studies have explored the role(s) of 27‐OHC in neurons and astrocytes. In the present study, to investigate the effects of 27‐OHC on inflammation in neurons and astrocytes, we tested the secretion of IL‐1β, IL‐10, TNF‐α, and iNOS, and measured the expression of toll‐like receptor 4 (TLR4) and transforming growth factor β (TGF‐β) and their downstream factors NF‐κB and COX‐2 in SH‐SY5Y and C6 cells treated with 27‐OHC.

## 2 MATERIALS AND METHODS

### 2.1 Cell lines and cell culture

SH‐SY5Y, a human neuroblastoma cell line, was obtained from the Cell Resource Center, IBMS, CAMS/PUMC, Beijing, China. The C6 rat glioma cell line was obtained from the Type Culture Collection of the Chinese Academy of Sciences, Shanghai, China. The compound, 27‐OHC (540 μg), was purchased from Santa Cruz Biotechnology, USA, and dissolved in 200 μl of dimethylsulfoxide (DMSO) to create a stock solution; the stock solution was then diluted in DMEM to 400 µM and stored at −80°C until use. Cells were cultured in DMEM containing 10% fetal bovine serum and streptomycin (10 U/L) and incubated in 5% CO_2_ at 37°C. All experiments were conducted under treatment with various treatments (5, 10, or 20 μM) of 27‐OHC or vehicle (DMEM) for 24 hr, and cells or culture media were then collected for the indicated assay measurements. SH‐SY5H and C6 cells were treated with DMEM or 27‐OHC (5, 10 or 20 μM) for 24 hr and harvested for measurement. The dose range of 27‐OHC was based on our previous study (An et al., 2017; Ma, Li et al., 2015).

### 2.2 Enzyme‐linked immunosorbent assay (ELISA)

SH‐SY5H and C6 cells (approximately 1 × 10^5^ cells/well) were treated with DMEM or 27‐OHC (5, 10 or 20 μM) for 24 hr. Supernatants from the cultures were collected and centrifuged at 800 *g* for 10 min. The levels of IL‐1β, TNF‐α, and iNOS were determined by using corresponding ELISA assay kit (Ray Biotech, USA) according to the manufacturer's instructions. The level of IL‐10 was also detected with an ELISA assay kit (United States Biological, USA). In both assays, absorbance at 450 nm was measured, and the levels of IL‐1β, TNF‐α, iNOS, and IL‐10 were calculated according to the standard curves.

### 2.3 Immunofluorescence methods

SH‐SY5Y and C6 cells were seeded into 6‐well plates with glass slips (approximately 2 × 10^4^ cells/well). After adhering to the coverslips, the cells were treated with 27‐OHC (0, 5, 10, and 20 μM) for 24 hr. The cells were then washed three times with PBS and then fixed in 4% phosphate‐buffered paraformaldehyde for 30 min. Next, the cells were permeabilized with phosphate‐buffered 0.1% Triton X‐100 for 20 min. The permeabilized cells were then incubated with fetal bovine serum (FBS) for 1 hr and subsequently incubated in primary rabbit anti‐TLR‐4 (Wuhan Servicebio Technology Company, China; 1:400 dilution) or rabbit anti‐TGF‐β (Wuhan Servicebio Technology Company, China; 1:400 dilution) overnight at 4°C, followed by incubation in goat anti‐rabbit CY3 secondary antibody (Wuhan Servicebio Technology Company, China; 1:300 dilution) at room temperature for 50 min. Immunolabeled cells were washed and mounted on gelatin‐coated slides using DAPI containing antifade mounting medium for 10 min. Then, DAPI was added to stain the cell nucleus for 10 min. Finally, the slips were examined for blue and red fluorescence by using a fluorescence microscope (Nikon, Japan). Image‐Pro Plus 6.0 was used to analyze the average fluorescence intensity.

### 2.4 Real‐time PCR analysis

The mRNA levels of NF‐κB p65, NF‐κB p50, and COX‐2 were determined by using quantitative real‐time PCR as previously described (Starr et al., 2011). Briefly, 1.0 μg of total RNA was reverse transcribed into cDNA using an M‐MLV reverse transcriptase kit (Thermo Scientific, USA). The expression of NF‐κB p65, NF‐κB p50, and COX‐2 mRNAs was quantified by real‐time PCR using an ABI real‐time PCR system (Applied Biosystems, USA) under standard qRT‐PCR conditions. Primer sequences are provided in Table 1. The calculation of mRNA was based on the 2^−ΔΔCT^ method with normalization to GAPDH.

**Table 1:** Primer sequences and fragment length of NF‐κB p65, NF‐κB p50 and COX‐2

Primer	Sense primer (5′–3′)	Antisense primer (5′–3′)	bp
GAPDH (h)	CATCTTCTTTTGCGTCGCCA	TTAAAAGCAGCCCTGGTGACC	115
NF‐κB p65 (h)	CTTCCTGCCCTACAGAGGTC	AAGGCACTTGAGAAGAGGGA	144
NF‐κB p50 (h)	GTGCAGAGGAAACGTCAGAA	GTGGGAAGCTATACCCTGGA	148
COX–2 (h)	AGCATCTACGGTTTGCTGTG	CCTGTTTAAGCACATCGCAT	114
GAPDH (r)	GGCCTTCCGTGTTCCTACC	CGCCTGCTTCACCACCTTC	103
NF‐κB p65 (r)	GGTTTGAGACATCCCTGCTT	TATGGCTGAGGTCTGGTCTG	92
NF‐κB p50 (r)	CATCCACCATGGAAGACAAG	CCAGCAGCATCTTCACATCT	138
COX–2 (r)	CACGGACTTGCTCACTTTGT	ACTGCTTGTACAGCGATTGG	92

Note

COX‐2: cyclooxygenase‐2; GAPDH: glyceraldehyde‐3‐phosphate dehydrogenase; h: human; NF‐κB p50: nuclear factor‐κB p50; NF‐κB p65: nuclear factor‐κB p65; r: rat.

### 2.5 Western blot analysis

SH‐SY5Y and C6 cells were treated with either vehicle or 27‐OHC (5, 10 or 20 μM) for 24 hr. Protein expression of NF‐κB p50, NF‐κB p65, and COX‐2 was measured by Western blot as previously described (Sui et al., 2009). Cell lysates were collected using RIPA buffer, and protein concentrations were determined using a BCA protein assay kit (Pierce Biotechnology, USA). Protein samples (50 μg/well) were loaded and separated with 12% SDS–polyacrylamide gel electrophoresis and wet transferred to a polyvinylidene fluoride (PVDF) membrane at a voltage of 60 V for 2 hr. Membranes were blocked with fresh blocking buffer (Tris‐buffered saline containing 5% skim milk powder) at room temperature for 1 hr. The membranes were then incubated with primary antibodies for NF‐κB p50 (1:1,000, Santa Cruz Biotechnology, USA), NF‐κB p65 (1:1,000, Abcam, USA), COX‐2 (1:1,000, Abcam, USA), and β‐actin (1:1,000, Cell Signaling, USA) overnight at 4°C, followed by incubation with corresponding secondary antibodies for 1 hr at room temperature. The blots were washed three times with TBST buffer, and protein bands were visualized by using an alkaline phosphatase reaction kit according to the manufacturer's instructions. The FluorChem FC_2_ software (Alpha Innotech, USA) was used to acquire images and quantify the grayscale value of each protein. The protein expression level for each gene was normalized to the level of β‐actin.

### 2.6 Statistical analysis

All data were analyzed by using the SPSS 18.0 software (IBM, USA). All data are presented as the mean ± standard deviation (*SD*) of at least three independent experiments. The means of different groups were compared by one‐way ANOVA followed by LSD or Tamhane's post hoc analysis. A two‐tailed *p* < 0.05 was considered statistically significant.

## 3 RESULTS

### 3.1 The effect of 27‐OHC on the secretion of IL‐1β, IL‐10, TNF‐α, and iNOS

SH‐SY5Y and C6 cells were treated with either DMEM or 27‐OHC (5, 10, or 20 μM) for 24 hr. The levels of secreted IL‐1β, IL‐10, TNF‐α, and iNOS in culture medium were then determined by ELISA. The levels of two pro‐inflammatory factors, TNF‐α (*p* = 0.033, Table 2) and iNOS (*p* < 0.01, Table 3), were significantly increased in SH‐SY5Y cells treated with 10 μM and 20 μM 27‐OHC, while the secretion of IL‐1β, another pro‐inflammatory cytokine, was not significantly changed in SH‐SY5Y cells in response to 27‐OHC. In contrast, the secretion of IL‐10, an anti‐inflammatory cytokine, was significantly decreased in SH‐SY5Y cells treated with 27‐OHC at 10 and 20 μM (both *p* < 0.05, Table 2) when compared to the control cells. Exposure of C6 cells to 10 and 20 μM 27‐OHC led to the significantly reduced secretion of IL‐1β (*p* = 0.008 and *p* < 0.01, respectively), IL‐10 (both *p* < 0.01), and TNF‐α (*p* = 0.027 and *p* = 0.008, respectively) in a dose‐dependent manner (Table 2). Secretion of iNOS from 10 and 20 μM 27‐OHC‐treated C6 cells was significantly increased (both *p* < 0.01, Table 3).

**Table 2:** IL‐1β, IL‐10, and TNF‐α levels in SH‐SY5Y and C6 cells after 27‐OHC treatment (mean ± *SD*)

	TNF‐α (pg/ml)		IL−1β (pg/ml)		IL−10 (pg/ml)	
	SH‐SY5Y	C6	SH‐SY5Y	C6	SH‐SY5Y	C6
Control	56.66 ± 4.04	99.36 ± 1.95	2.46 ± 1.13	60.20 ± 5.77	18.92 ± 0.65	35.00 ± 1.88
27‐OHC 5 μM	58.49 ± 2.58	95.60 ± 2.45	1.79 ± 0.97	58.87 ± 7.53	18.61 ± 1.20	34.33 ± 2.03
27‐OHC 10 μM	134.73 ± 13.07^a^, ^b^	91.03 ± 5.16^a^	1.37 ± 0.63	40.86 ± 9.53^a^, ^b^	11.82 ± 0.49^a^, ^b^	21.14 ± 1.41^a^, ^b^
27‐OHC 20 μM	62.17 ± 1.00	88.69 ± 4.50^a^	1.06 ± 0.71	29.15 ± 2.10^a^, ^b^	10.91 ± 0.44^a^, ^b^	7.62 ± 0.27^a^, ^b^, ^c^

Note

IL‐1β, IL‐10, and TNF‐α levels in SH‐SY5Y cells treated with vehicle (control group), 5, 10, or 20 µM 27‐OHC. All data were presented as mean ± *SD* of three independent experiments.

IL‐1β: interleukin‐1β; IL‐10: interleukin‐10; TNF‐α: tumor necrosis factor alpha.

a Mean value was significantly different from that of the control group, *p* < 0.05.

b Mean value was significantly different from that of the 5 μM 27‐OHC group, *p* < 0.05.

c Mean value was significantly different from that of the 10 μM 27‐OHC group, *p* < 0.05.

**Table 3:** iNOS level in SH‐SY5Y and C6 cells in different 27‐OHC‐treated groups

	iNOS (ng/ml) SH‐SY5Y	iNOS (ng/ml) C6
Control	3.44 ± 0.03	7.95 ± 0.15
27‐OHC 5 μM	3.53 ± 0.10	8.13 ± 0.53
27‐OHC 10 μM	3.57 ± 0.08	10.19 ± 0.32^a^, ^b^
27‐OHC 20 μM	3.99 ± 0.02^a^, ^b^	10.57 ± 0.29^a^, ^b^

Note

iNOS level in SH‐SY5Y cells treated with vehicle (control group), 5, 10, or 20 µM 27‐OHC. All data were presented as mean ± *SD* of three independent experiments. iNOS: inducible nitric oxide synthase.

a Mean value was significantly different from that of the control group, *p* < 0.05.

b Mean value was significantly different from that of the 5 μM 27‐OHC group, *p* < 0.05.

c Mean value was significantly different from that of the 10 μM group, *p* < 0.05.

### 3.2 The effects of 27‐OHC on the expression of TLR‐4 and TGF‐β

**Figure 1:**
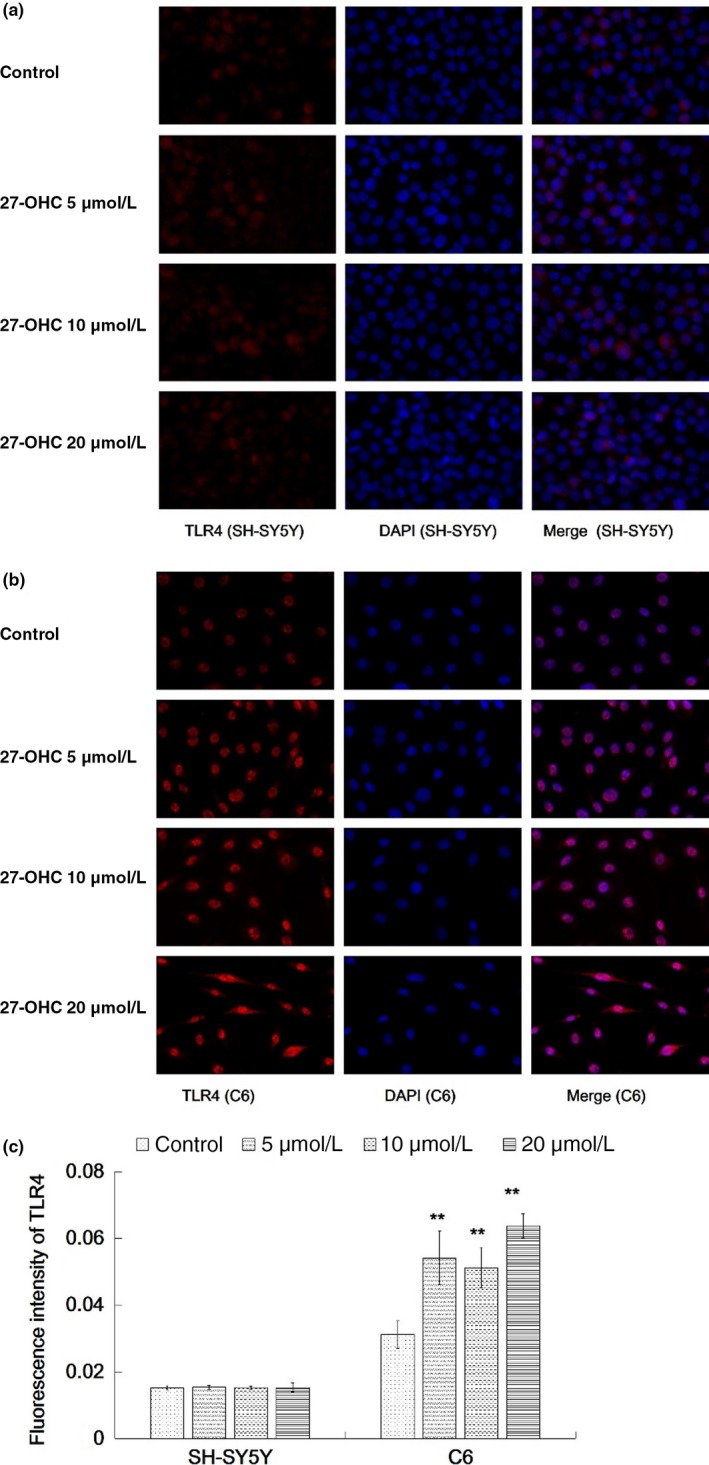
TLR4 expression was upregulated in C6 but not SH‐SY5Y cells in response to 27‐OHC treatment. SH‐SY5Y and C6 cells were treated with DMEM (control), or 5, 10 or 20 μM 27‐OHC for 24 hr. (a) TLR4 expression in SH‐SY5Y cells were measured with immunofluorescence staining. (b) TLR4 expression in C6 cells was measured with immunofluorescence staining. (c) Quantification of the immunofluorescence images. Data represented mean ± *SD* of three independent experiments. Mean value was significantly different from that of the control group **p < 0.01. TLR4: toll‐like receptor 4

**Figure 2:**
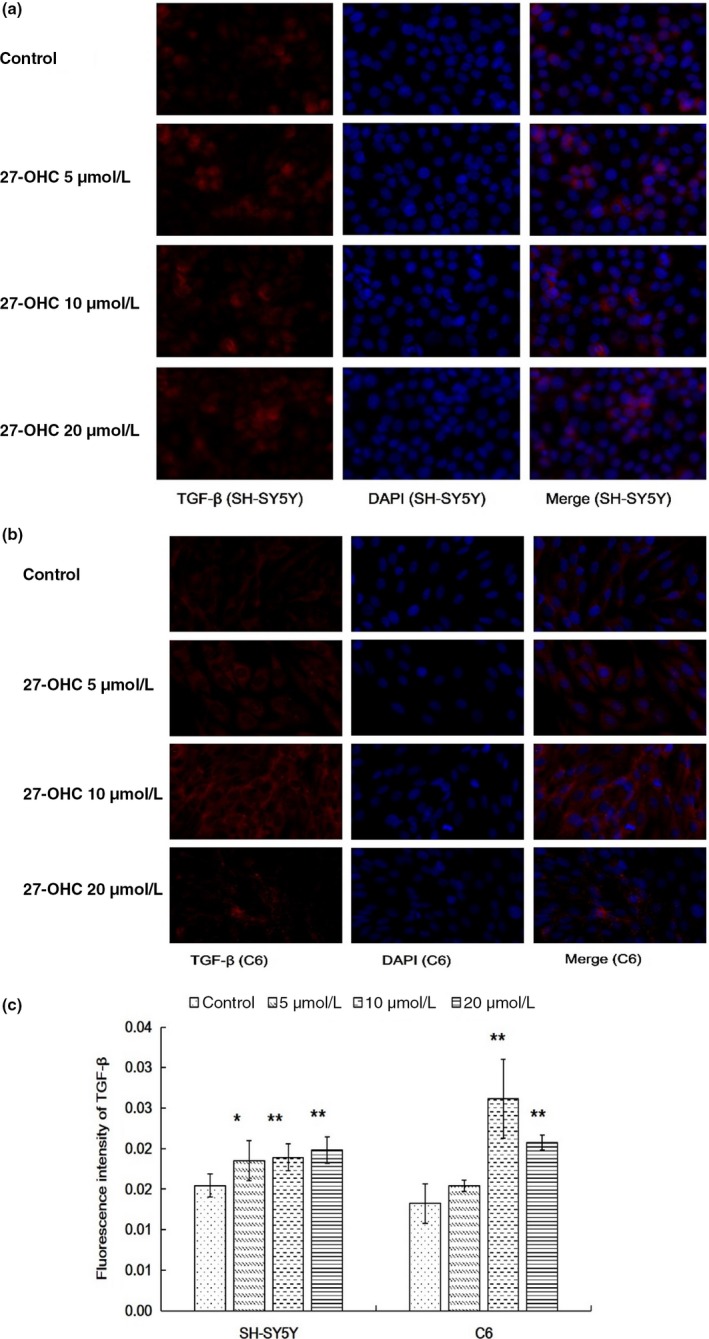
TGF‐β expression was increased in both SH‐SY5Y and C6 cells treated with 27‐OHC. SH‐SY5Y and C6 cells were treated with DMEM (control), or 5, 10 or 20 μM 27‐OHC for 24 hr. (a) TGF‐β expression in SH‐SY5Y cells was measured with immunofluorescence staining. (b) TGF‐β expression in C6 cells was measured with immunofluorescence staining. (c) Quantification of the immunofluorescence images. Data represented mean ± *SD* of three independent experiments. Mean value was significantly different from that of the control group *p < 0.05; **p < 0.01. TGF‐β: transforming growth factor‐β

Immunofluorescence staining showed that exposure to 27‐OHC at 5, 10, and 20 μM did not lead to significant alterations in TLR4 expression in SH‐SY5Y cells (all *p* > 0.01, Figure 1a,c). In contrast, all three concentrations of 27‐OHC resulted in upregulated expression of TLR4 in C6 cells (all *p* < 0.01, Figure 1b,c). Furthermore, incubation with 27‐OHC (5, 10 and 20 μM) significantly increased the expression of TGF‐β in SH‐SY5Y cells (Figure 2a,c), similar results were observed in C6 cells exposed to 10 and 20 μM 27‐OHC (all *p* < 0.05, Figure 2b,c).

### 3.3 The effect of 27‐OHC on NF‐κB p65, NF‐κB p50, and COX‐2 mRNA expression

**Figure 3:**
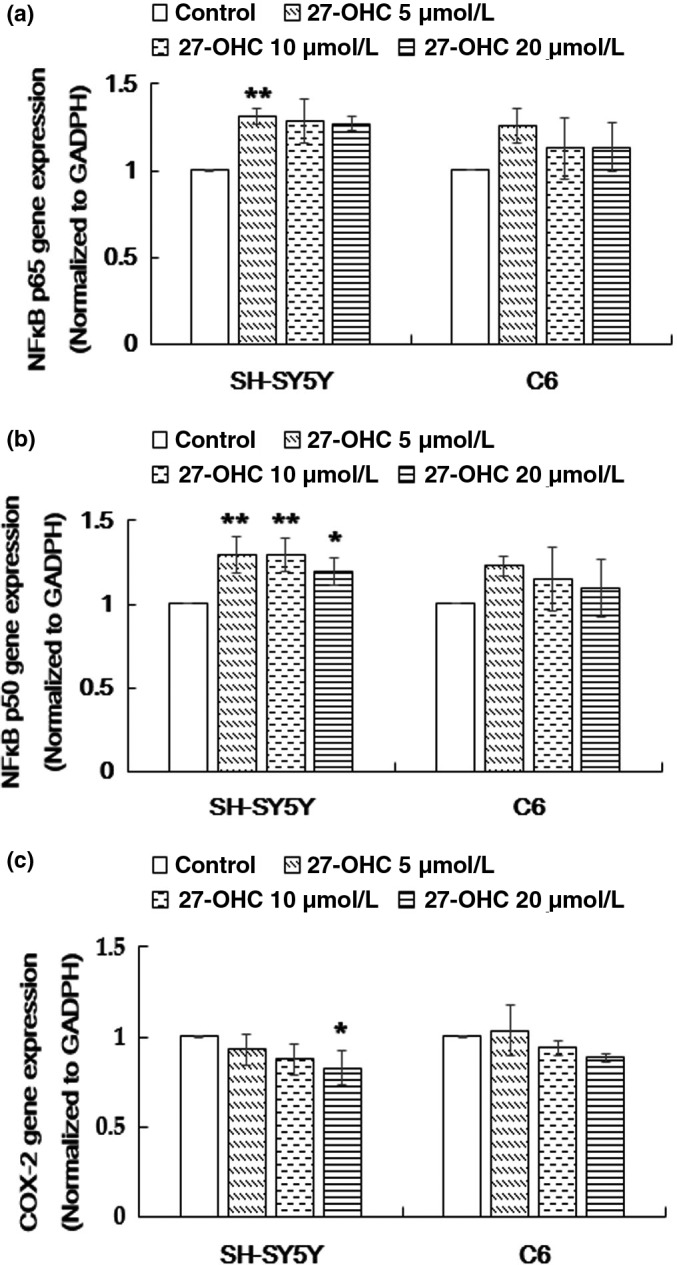
The effect of 27‐OHC on NF‐κB p65, NF‐κB p50, and COX‐2 mRNA expression. SH‐SY5Y and C6 cells were treated with DMEM (control), or 5, 10 or 20 μM 27‐OHC for 24 hr. (a) The mRNA expression of NF‐κB p65 was measured with qRT‐PCR. (b) The mRNA expression of NF‐κB p50 was measured with qRT‐PCR. (c) The mRNA expression of COX‐2 was measured with qRT‐PCR. Data represented mean ± *SD* of three independent experiments. Mean value was significantly different from that of the control group *p < 0.05; **p < 0.01. COX‐2: cyclooxygenase‐2; NF‐κB p65: nuclear factor‐κB p65; NF‐κB p50: nuclear factor‐κB p50

As shown in Figure 3b, the mRNA level of NF‐κB p50 was upregulated in SH‐SY5Y cells in response to 27‐OHC at 5, 10, and 20 µM (*p* = 0.003, *p* = 0.003, and *p* = 0.024, respectively), while the mRNA expression of NF‐κB p65 was only upregulated in cells treated with 5 μM 27‐OHC (*p* = 0.046, Figure 3a). The expression of COX‐2 was significantly inhibited in SH‐SY5Y cells by 20 µM 27‐OHC treatment (*p* = 0.023, Figure 3c). However, no significant differences in mRNA levels of NF‐κB p65, NF‐κB p50, and COX‐2 were found between the control and 27‐OHC‐treated C6 cells (all *p* > 0.05, Figure 3a‐c).

### 3.4 27‐OHC did not alter NF‐κB p65, NF‐κB p50, or COX‐2 protein expression

**Figure 4:**
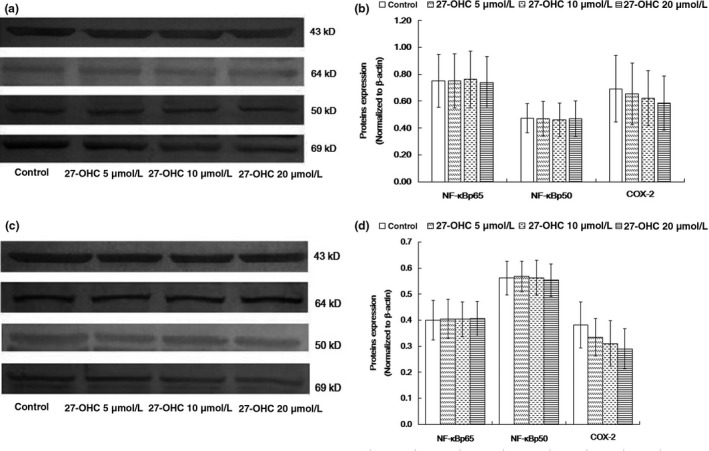
27‐OHC did not alter NF‐κB p65, NF‐κB p50, and COX‐2 protein expression. SH‐SY5Y and C6 cells were treated with DMEM (control), or 5, 10 or 20 μM 27‐OHC for 24 hr. The protein expression of NF‐κB p65, NF‐κB p50, and COX‐2 was determined by Western blot. (a) Representative immunoblots of SH‐SY5Y cells. (b) Quantification of panel a. (c) Representative immunoblots of C6 cells. (d) Quantification of panel c. Data represented mean ± *SD* of three independent experiments. COX‐2: cyclooxygenase‐2; NF‐κB p65: nuclear factor‐κB p65; NF‐κB p50: nuclear factor‐κB p50

The protein expression levels of NF‐κB p65, NF‐κB p50, and COX‐2 were not significantly altered in both SH‐SY5Y and C6 cells exposed to different concentrations of 27‐OHC, when compared with the protein expression levels of the control cells (all *p* > 0.05, Figure 4a‐d).

## 4 DISCUSSION

Studies have demonstrated that Aβ accumulates in the gray matter of brains from AD patients and is surrounded by a large number of activated microglia and astrocytes (Fakhoury, 2018), which secrete a series of inflammatory factors such as IL‐l, IL‐6, IL‐8, and TNF‐α (Chen et al., 2017). Therefore, overactivation of inflammatory responses in the brain may represent an important etiology for AD (Yin, Sancheti, Patil, & Cadenas, 2016). IL‐l in the brain is produced by glial cells, endothelial cells, and neurons, which have two subtypes: IL‐1α and IL‐1β. IL‐1α is mainly expressed on the inner side of the plasma membrane, while IL‐1β is secreted extracellularly. Excessive expression of IL‐1β is closely related to AD; this close relation is because low levels of IL‐1β promote the growth of neurons and astrocytes, while high levels of IL‐1β may be toxic to neurons and astrocytes (Ali et al., 2013; Chen et al., 2018).

The metabolite 27‐OHC has been reported to promote the generation of Aβ in neurons through the increased expression of IL‐1β synthetic receptors (Gamba et al., 2011). IL‐1β may reduce the generation of Aβ1‐42 in cultured primary neurons by silencing astrocytes. Therefore, abnormally upregulated IL‐1β levels likely increase the accumulation of Aβ and eventually lead to the development of AD. The present study shows that the IL‐1β level was not markedly altered in neurons treated with different concentrations of 27‐OHC; however, IL‐1β was downregulated in a dose‐dependent manner in astrocytes treated with 27‐OHC for 24 hr. This downregulation may indicate that IL‐1β was increased as an acute inflammatory factor in the brain at early time points in response to 27‐OHC stimulation, but cell damage was aggravated after 24 hr when the levels of IL‐1β were decreased. Rosklint, Ohlsson, Wiklund, Noren, and Hulten (2002) reported that 27‐OHC dose‐dependently increased the expression of IL‐1β in macrophages stimulated with lipopolysaccharide (LPS), but upregulation of IL‐1β was not stable without this 27‐OHC‐LPS costimulation. In the present study, IL‐1β was increased in SH‐SY5Y cells in response to 27‐OHC during the first 24 hr; however, these changes were not detectable beyond 24 hr, suggesting that the release of inflammatory factors may occur in the early stages of the inflammatory reaction.

As an anti‐inflammatory factor, IL‐10 may inhibit the activation of macrophages and microglia and reduce the synthesis of cytokines. Lee, McGeer, and McGeer (2015) reported that IL‐10 could reduce the expression of tau protein in microglia that was stimulated by LPS. However, IL‐10 did not seem to play the same role in SH‐SY5Y cells. IL‐10 inhibited the synthesis and release of IL‐1, IL‐6, and TNF‐α in neurons stimulated with Aβ1‐42 (Hawkins, MacKay, MacKay, & Moldawer, 1998). In this study, treatment with 27‐OHC resulted in an inflammatory response in neurons and astrocytes and decreased the level of the anti‐inflammatory factor IL‐10.

Tumor necrosis factor alpha is widely expressed in microglia, astrocytes, macrophages, endothelial cells, and neurons. An unnecessary increase in TNF‐α is reported to be toxic to neurons. Reinsfelt, Westerlind, Blennow, Zetterberg, and Ricksten (2013) reported that increased levels of TNF‐α in serum and cerebrospinal fluid were related to the levels of Aβ. The amount of TNF‐α in the brain was increased in AD rats, and the mRNA expression of TNF‐α in the hippocampus was also significantly increased as well (Solmaz et al., 2015). These results indicate that TNF‐α likely participates in the pathogenesis of AD. The metabolite 27‐OHC has been shown to activate the phosphorylation of tau protein and upregulate the expression of TNF‐α in the hippocampus of rabbits (Prasanthi, Larson, Schommer, & Ghribi, 2011). Our study shows that 27‐OHC increased TNF‐α secretion in neurons but decreased its secretion in astrocytes. This result suggests that increases in TNF‐α expression may occur earlier in astrocytes than in neurons. Therefore, severe inflammatory reactions have already occurred in C6 cells after treatment with 27‐OHC for 24 hr, and these subsequent inflammatory reactions may result in apoptosis.

Under pathological conditions, iNOS is found at increased levels in the central nervous system. Upregulated iNOS leads to the release of ROS, which can cause serious damage to neurons. Expression of iNOS is assumed to activate glial cells and reflects the extent of lipid peroxidation and generation of ROS in the body (Chen et al., 2017). For example, the expression of iNOS was significantly higher in the rat pheochromocytoma PC12 cells than in normal cells (Kim et al., 2015). Our study indicates that 27‐OHC can increase the expression of iNOS in both neurons and astrocytes, thus suggesting that 27‐OHC may cause inflammation in neurons and astrocytes through iNOS‐mediated mechanisms.

Toll like receptor 4 is recognized as a component of the primary innate immune receptor‐mediated inflammatory signaling pathway (Li et al., 2018). Researchers demonstrated that the functional activation of TLR4 is essential for increased TGF‐β mRNA expression in response to 27‐OHC in epithelial cells (Pei, Lin, Song, Li, & Yao, 2008). In our study, we investigated the effect of 27‐OHC on the expression of TLR4 and TGF‐β in SH‐SY5Y cells and C6 cells. The results showed that 27‐OHC treatment increased the expression of TGF‐β in SH‐SY5Y cells and increased the expression of both TLR4 and TGF‐β in C6 cells, which indicates that 27‐OHC has different effects on inflammatory factor production in neurons and astrocytes.

NF‐κB is widely distributed throughout the nervous system and is involved in the inflammation, cell proliferation, and apoptosis. It has been reported that TGF‐β causes an increase in NF‐κB binding to DNA in the absence of NF‐κB translocation (Hogan, Ravindran, Podolsky, & Glick, 2013). Moreover, NF‐κB increases the expression levels of a series of inflammatory cytokines and enzymes, such as IL‐6, IL‐1, iNOS, and COX‐2. When NF‐κB is normally expressed, it plays a protective role in neurons, but excessive activation of NF‐κB can result in the release of a large amount of inflammatory factors in glial cells, and subsequent acceleration of the development of AD. NF‐κB mainly exists in the forms of p65 and p50. COX‐2 is a membrane‐bound protein that stimulates the production of inflammatory mediators, cytokines and complements and is involved in cell proliferation, immune responses and signal transduction. NF‐κB regulates COX‐2 expression through transcriptional regulation. Increased expression of COX‐2 in the brains of AD patients is highly associated with the level of Aβ. Overexpression of COX‐2 in a neuron can damage other neurons directly or indirectly by increasing the activity of inflammatory signaling pathways, ultimately leading to the death of the neurons. (Kikuchi et al. (2012) reported that NF‐κB levels in the lung and phlegm of patients with chronic obstructive pulmonary disease were significantly increased when compared to levels in the lung and phlegm of healthy controls. The metabolite 27‐OHC has been reported to activate the transcription of NF‐κB and increase the expression of downstream TGF‐β in muscle cells. According to the results of this study, 27‐OHC may increase the mRNA expression of p65 and p50 and decrease the expression of COX‐2 mRNA in neurons but not in astrocytes. However, no obvious changes in the protein expression levels of NF‐κB p65, p50, and COX‐2 were observed in neurons and astrocytes treated with 27‐OHC. Our study suggests that 27‐OHC induces inflammatory damage to neurons but not astrocytes, possibly through the activation of the NF‐κB signaling pathway.

In summary, 27‐OHC induces inflammatory damage to neurons, likely through the activation of the TGF‐β/NF‐κB signaling pathway. However, treatment with 27‐OHC in astrocytes resulted in inflammatory damage through the activation of TLR4/TGF‐β signaling pathway and the subsequent release of inflammatory cytokines.

## CONFLICT OF INTEREST

The authors declare that they do not have any conflict of interest.

## ETHICAL STATEMENTS

This study does not involve any human or mammal testing.
